# Characterization and Performance Analysis of Underwater Optical Time and Frequency Dissemination Link Based on Monte Carlo Simulation and Experimental Demonstration

**DOI:** 10.3390/s25226861

**Published:** 2025-11-10

**Authors:** Yibo Yuan, Hengrui Liu, Ziyi Wang, Hanwen Zhang, Xujin Li, Jianfeng Cui, Yiguang Yang

**Affiliations:** 1 College of Ocean Science and Engineering, Shandong University of Science and Technology, 579 Qianwangang Road, Qingdao 266590, China; luckyyuanyibo@163.com (Y.Y.); 202383190036@sdust.edu.cn (H.L.); 202483190064@sdust.edu.cn (H.Z.); 2 School of Computer and Communication Engineering, University of Science and Technology Beijing (USTB), Beijing 100083, China; 18753790357@163.com; 3 Laoshan Laboratory, Qingdao 266237, China; xjli@qnlm.ac; 4 Anshan ZY Laser Technology Co., Ltd., Anshan 114041, China; cuijf@163.com

**Keywords:** underwater wireless optical communication, underwater optical time–frequency transfer, time–frequency synchronization

## Abstract

Underwater Wireless Optical Communication (UWOC) plays a crucial role in marine exploration and observation due to its high speed and low latency characteristics, while research on underwater time and frequency transfer (UTFT) is relatively lacking. The complicated underwater environments, absorption and scattering effects severely degrade signal stability and signal-to-noise-ratio (SNR). In response to this issue, a photon packet transmission model is established based on the Monte Carlo simulation (MCS). The effects of different parameters, including water conditions, divergence angles, receiving apertures, are systematically analyzed, with key indicators such as phase noise and Allan deviation, identified as performance measures. An experimental platform is also built using kaolin turbidity to obtain experimental results corresponding to different frequencies and turbidity levels, which are then compared with simulation results. The high consistency between simulation and experimental results verifies the reliability of the proposed model. This research provides a feasible method for performance prediction and tolerance design of UTFT networks.

## 1. Introduction

In modern marine exploration and observation, underwater communication has become an indispensable and crucial technology. In recent years, underwater time–frequency synchronization (TFS) via underwater time and frequency transfer (UTFT) has become of great importance [1], Autonomous Underwater Vehicles (AUVs) and underwater positioning and navigation are highly dependent on high-precision TFS. During operation, they must rely on TFS to share timing data, thereby preventing the failure of data fusion algorithms and chaos in task coordination [2,3,4]. Underwater positioning systems such as Long Baseline (LBL) are implemented by measuring the Time Difference of Arrival (TDOA) between different base stations. If clock asynchrony occurs, it will lead to positioning errors or even complete system failure. In the UTFT link, signals may be subjected to various interferences, which can lead to TFS issues and hinder the normal operation of equipment [5,6].

Although the TFS based on acoustic sonar has exhibited good results, due to relatively low frequencies and low propagation speeds of the acoustic characteristics, even small relative movements can lead to significant Doppler frequency shifts. Its limited bandwidth results in high propagation delays, so the research on TFS underwater acoustic signals has certain limitations [7]. Severe multipath effects in the underwater environment and long-term operation of the system may all cause errors such as clock drift, synchronization failure, and frame alignment issues, posing greater challenges to underwater acoustic TFS [8,9].

In recent years, Underwater Wireless Optical Communication (UWOC) has been widely applied. Lasers used in UWOC, especially those operating in the blue-green wavelength band, exhibit low absorption coefficients. Consequently, as an emerging and promising technology for high-speed underwater wireless communication, UWOC has exhibited pioneering development [10,11]. Compared with underwater acoustic communication, UWOC has demonstrated significant progress in aspects such as low latency, high-rate transmission, and enhanced anti-interference capability [12,13,14], has attracted attention in both the academic research community and industrial applications [15]. To address challenges such as signal attenuation, turbulence, and temperature variations caused by the complicated underwater environment, researchers have proposed a variety of technical approaches, including communication link design [16,17], bandwidth optimization [18,19], and pulse modulation [20]. It is the maturity of underwater optical communication technology that enables underwater optical TFS. In recent years, a large number of studies on UWOC have been conducted using numerical simulation and experimental methods [21], while there has been relatively little research on underwater optical TFS. Relevant research has proposed technologies such as optical phase compensation, which provide effective solutions for underwater frequency transmission [22]. However, the UTFT still faces interferences such as turbulence and the Doppler effect, and the optimization of its performance remains an urgent issue to be addressed at present.

In the field of optical communications, the Monte Carlo simulation (MCS) method is widely used to simulate the effects of various random factors such as atmospheric turbulence [23], scattering, and absorption [24]. Owing to its basis on probabilistic modeling, its application in addressing complex systems and multi-dimensional problems demonstrates significant flexibility and applicability. In multipath propagation simulation, it is often employed to model the photon propagation and scattering processes [25]. Consequently, MCS can effectively simulate the channel state of UTFT and provide simulation conclusions that closely accord with real-world conditions for certain modeling approaches [26].

This paper introduces the MCS method to simulate the UTFT. Considering the differences in signal quality under different transmission states across various water conditions, this study comprehensively analyzes the influencing factors and characteristics of the signal. Through systematic experiments, we analyzed the relationship between the phase noise characteristic parameters and different turbidity levels, and proposed a linear characterization model, which provides a new approach for optimizing the TFS performance.

This paper is structured as follows: Section 1 introduces the research background of UTFT, highlighting the limitations of existing acoustic-based methods and the potential of UWOC for UTFT. Section 2 details the MCS-based photon packet transmission model, including key parameter definitions, scattering/absorption modeling, and the experimental setup with kaolin turbidity. Section 3 presents and analyzes simulation and experimental results, focusing on the impacts of water conditions, system parameters, and signal frequency on UTFT performance. Section 4 summarizes the core contributions, and the final part outlines future research directions to promote UTFT practicalization.

## 2. Theoretical Foundation of the Method

**Figure 1:**
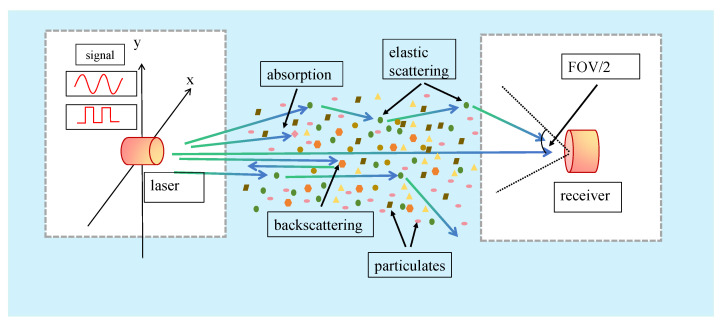
Seawater photon packet MCS channel model.

When laser light travels through seawater, the attenuation of photons is mainly caused by suspended particles and dissolved organisms. Scattering alters the propagation path of photons, resulting in attenuation and distortion of the signal at the receiving end. The attenuation of the light beam is the combined effect of absorption and scattering effects. The simulated seawater channel MCS model is illustrated in Figure 1.

The effects of absorption and scattering on photon propagation in seawater are denoted by α and β, respectively. The total effect on photon energy is represented by the extinction coefficient (also referred to as the beam attenuation coefficient) *c*, where c=α+β. Meanwhile, taking into account the factors of light attenuation, the propagation of a light beam through seawater over a path length L results in an exponential attenuation of its intensity, described by the following: $I\left(L\right)=I_{0}exp\left[-\left(\alpha +\beta \right)L\right]$. In this expression, $I_{0}$ represents the initial light intensity at the source, and I(L) is the intensity remaining after traversal of the distance *L*. The exponential factor, exp[−(α+β)L], quantifies the collective attenuation effect, where the absorption coefficient (α) and the scattering coefficient (β) jointly determine the rate at which the light intensity diminishes.

The values of scattering, absorption, and extinction coefficients in different types of seawater selected in this simulation are presented in Table 1. In this study, three typical water environments, namely clear water, coastal water, and harbor water, were selected.

**Figure 2:**
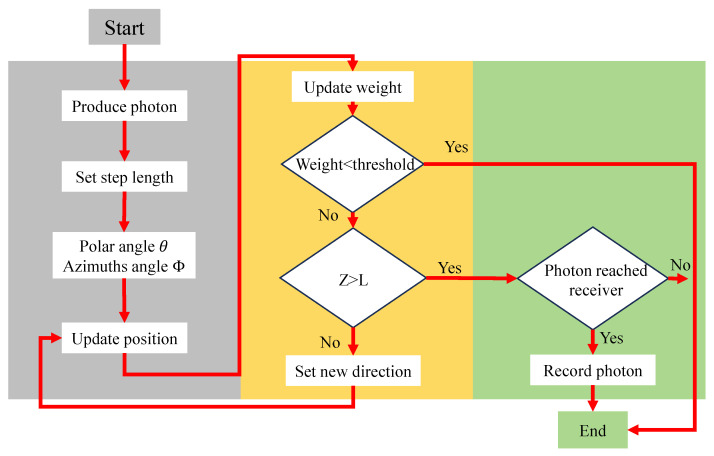
System flowchart of the MCS of photon packet propagation.

In this paper, the flowchart of the MCS for photon packet transmission is shown in Figure 2. Firstly, the laser generates and emits $10^{7}$ photon packets. The coordinate position of each photon packet is represented in the Cartesian coordinate system (*x*, *y*, *z*) with the initial position at (0, 0, 0). Each photon packet propagates independently, and during the photon packet emission process, a random step size is set:

(1) $$l=-\frac{ln(\zeta )}{c}.$$

where, *c* is the beam attenuation and ζ is a random number ranging from 0 to 1. After a photon packet scatters at direction cosines ($\mu_{x},\mu_{y},\mu_{z}$), the system updates its direction cosines ($\mu_{x}^{\prime },\mu_{y}^{\prime },\mu_{z}^{\prime }$), as shown in Equation (2), they describe the direction of propagation in terms of the polar angle θ and the azimuthal scattering angle ϕ=2πζ,

(2) $$\begin{array}{c} \begin{array}{c} \mu_{x}^{\prime }=\frac{sin\theta }{1-\mu_{z}^{2}}\left(\mu_{x}\mu_{z}cos\phi -\mu_{y}sin\phi \right)+\mu_{x}cos\theta \\ \mu_{y}^{\prime }=\frac{sin\theta }{1-\mu_{z}^{2}}\left(\mu_{y}\mu_{z}cos\phi -\mu_{x}sin\phi \right)+\mu_{x}cos\theta \\ \mu_{z}^{\prime }=-sin\theta cos\phi \sqrt{1-\mu_{z}^{2}}+\mu_{z}cos\theta . \end{array} \end{array}$$

The Henyey-Greenstein (H-G) phase function is used to describe the scattering direction distribution along the photon packets transport path, and its expression is given as follows:

(3) $$P_{HG}\left(\theta \right)=\frac{1-g^{2}}{4\pi (1+g^{2}-2gcos\theta {)}^{\frac{3}{2}}},$$

where θ is the scattering polar angle and *g* is the asymmetry factor of the H-G phase function, according to Equation (3), the expression for the scattering angle can be obtained by calculation as follows:

(4) $$\theta =arccos\{\frac{1}{2g}\left[1+g^{2}-{\left(\frac{1-g^{2}}{1-g+2g\zeta }\right)}^{2}\right]\}.$$

The forward scattering characteristic of seawater is remarkably prominent. This phenomenon is primarily ascribed to suspended particles and dissolved organisms in seawater, whose dimensions are generally larger than the laser wavelength, inducing a strong scattering bias in the small-angle regime. Consequently, in several classical marine optics studies, when establishing a model for laser propagation in seawater, the value of the forward scattering coefficient *g* is approximately set to 0.924 [21].

As illustrated in Figure 2, the simulation follows a step-wise process. Each photon packet is assigned an initial weight, random propagation direction, and path length determined by the medium scattering coefficient, with the cumulative transmission distance initialized simultaneously. The photon packet updates its position along a random direction, and its weight undergoes continuous attenuation during propagation. If the residual weight of a photon packet falls below a preset threshold, the simulation process is terminated; otherwise, it continues to propagate. In each propagation step, the cumulative transmission distance is updated accordingly. When a photon packet reaches the detector region (i.e., propagation depth Z≥L), the program calculates its position on the receiving plane. If the photon packet falls within the receiver aperture and lies within the field of view (FOV) of the detection system, its arrival time and attenuated amplitude are recorded. For non-terminated photon packets, their propagation direction undergoes random scattering according to the H-G phase function before proceeding to the next propagation cycle. This loop executes continuously until all photon packets complete their propagation paths, with the arrival times, received amplitudes, and scattering information of all photon packets recorded for subsequent statistical and phase analysis.

## 3. Simulation and Experiment Results

Based on the aforementioned flowchart and simulation configuration, MCS were performed for UTFT to investigate the impacts of different parameters (e.g., seawater type, divergence angle, receiving radius, and receiving FOV) on signal quality, and corresponding simulation conclusions were derived.

**Figure 3:**
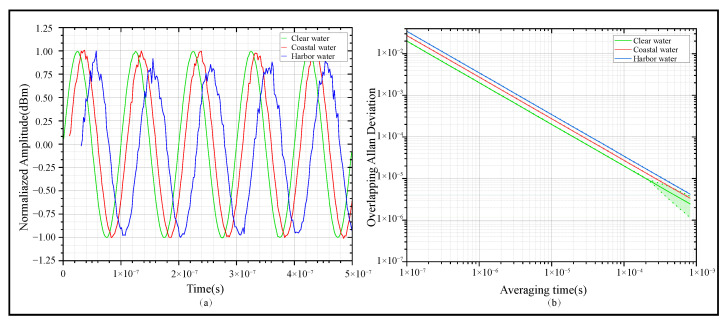
The influence of Mie scattering in different water bodies on frequency signal quality at a frequency of 1 MHz. (**a**) Signals across different water bodies. (**b**) Allan deviation of received signals across different water bodies.

In UTFT, the optical properties of the water body exert a decisive influence on the propagation of optical signals. In the simulation, the MCS was employed to model the photon packet propagation process under Mie scattering conditions, with the transmission distance set to 10 m. A Gaussian beam was used, and the normalized signal intensity at the receiving end was analyzed. The results are presented in Figure 3.

As illustrated in Figure 3a, for receiving signals related to different water bodies, the normalized peak intensities of clear water, coastal water, and harbor water are 1.0, 0.85, and 0.65, respectively, indicating that the signal energy decreases significantly with the increase in turbidity. As shown in Figure 3b, the Allan deviation curve rises with the increasing turbidity of water; the error bars are presented in the form of a light-color shaded area, whose corresponding confidence factor is 0.95. This phenomenon is associated with the large number of suspended particles and strong Mie scattering.

**Figure 4:**
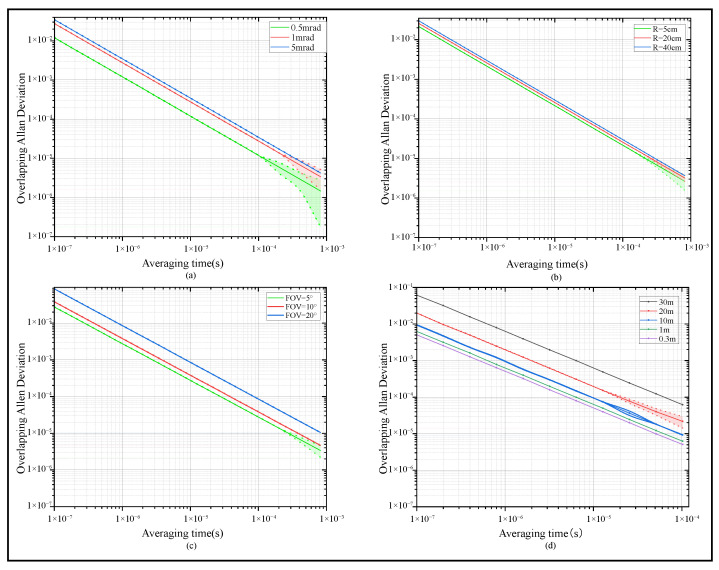
Allandeviation of receiving frequency signal corresponding to different system parameters. (**a**) Different transmitting divergence angles. (**b**) Different receiving apertures. (**c**) Different receiving FOV. (**d**) Different transfer distance.

Figure 4 presents the Allan deviation of the signal at the MCS receiving end under different system parameter conditions. Figure 4a–d correspond to independent simulation experiments, with all simulations conducted under coastal water conditions. As illustrated in Figure 4a, the Allan deviation exhibits a positive correlation with the divergence angle, showing that the signal stability decreases remarkably. To investigate the relationship of receiving aperture and FOV with signal quality, a simulation was conducted to analyze their effects on signal quality when other parameters were held constant. Specifically, receiving apertures of 5 cm, 20 cm, and 40 cm were selected as the variable in Figure 4b; FOVs of 5°, 10°, and 20° were selected as the variable in Figure 4c. As shown in Figure 4b,c, after photon packets undergo multipath scattering, which results in photons deviating from the main optical axis, different apertures and FOVs exhibit distinct performance. A smaller aperture or FOV better blocks the excessively scattered photon packets, ensuring the received signal mainly comes from photons near the main optical axis, thus boosting signal quality by filtering noise and improving signal purity. In contrast, a larger aperture or FOV allows more noise-inducing scattered photons to enter, increasing signal processing complexity and significantly reducing SNR. Meanwhile, aperture or FOV selection must consider signal intensity: overly small sizes may filter out useful photons, leading to insufficient signal intensity and compromising subsequent detection. Transferring distances of 0.3 m, 1 m, 10 m, 20 m, and 30 m were selected as the variables in Figure 4d. As the transferring distance increases, the Allan deviation rises significantly, indicating a gradual decline in the temporal stability of the signal. After photons enter the receiving end after multiple scatterings, the randomness of their paths and the effect of noise accumulation become more prominent as the distance increases. A longer distance leads to signal attenuation and a lower SNR, which in turn causes greater relative fluctuations and instability of the signal. This manifests as an increase in the Allan deviation at last.

**Figure 5:**
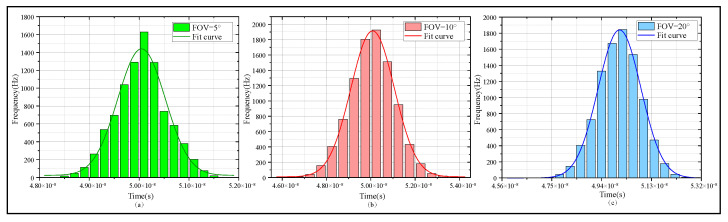
Histogram of the signal’s half-period time interval statistical results. (**a**) Histogram of signal’s half-period time interval statistical result when the FOV is 5°. (**b**) Histogram of signal’s half-period time interval statistical result when the FOV is 10°. (**c**) Histogram of signal’s half-period time interval statistical result when the FOV is 20°.

Histogram analysis was also performed for these parameters. Taking FOV as an example, others will not be enumerated one by one here. Figure 5 presents the histogram of the signal’s half-period time interval statistical results obtained from the FOV simulation.

The histogram in Figure 5 shows that a smaller FOV results in a denser distribution of central photon packet counts and fewer side-scattered photon packets. In contrast, a wider peak width leads to poor signal quality, strong noise, and difficulty in demodulating the received signal. It can be concluded that limiting the FOV size can effectively filter scattered photon packets and improve the relative signal quality. However, although reducing the FOV can enhance the SNR, it significantly weakens the received signal intensity. This issue is particularly prominent in scenarios with a large divergence angle, where it may cause the loss of photon packets. The MSC model proposed in this paper can provide references for the design of UTFT systems.

**Figure 6:**
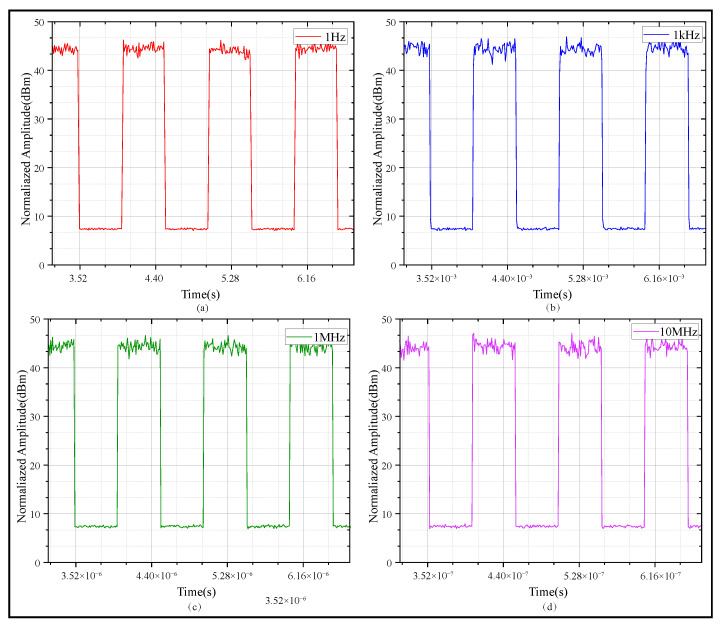
Simulated square wave signals with different frequencies. (**a**) 1 Hz square wave signal. (**b**) 1 kHz square wave signal. (**c**) 1 MHz square wave signal. (**d**) 10 MHz square wave signal.

**Figure 7:**
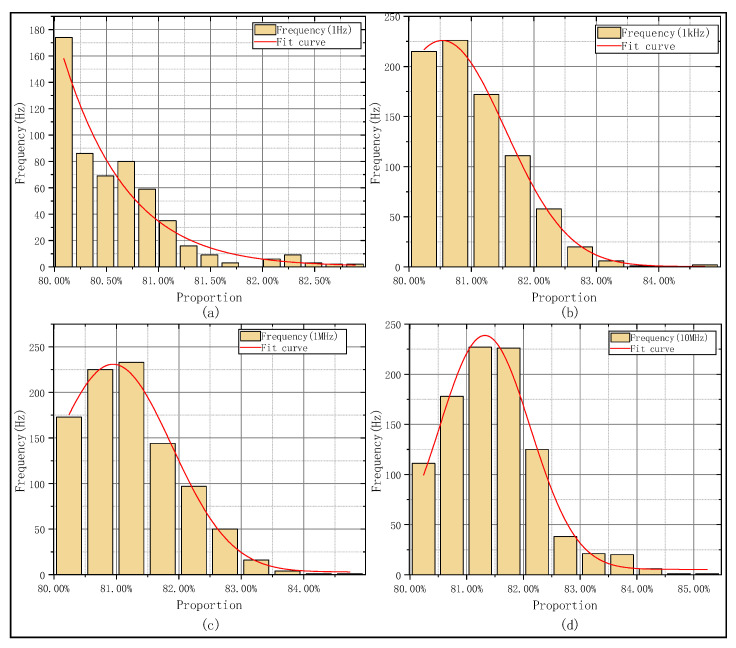
Histograms and fitting trends of 10–90% rising edge time proportion for time signals with different frequencies. (**a**) Rising edge time statistics of 1 Hz time signal. (**b**) Rising edge time statistics of 1 kHz time signal. (**c**) Rising edge time statistics of 1 MHz time signal. (**d**) Rising edge time statistics of 10 MHz time signal.

Afterwards in this section, square wave signals are employed to study the influence of water body scattering on time synchronization signals of different frequencies. First, four ideal square wave signals with frequencies of 1 Hz, 1 kHz, 1 MHz, and 10 MHz were generated in this study, with a rising edge set to 1 ns. The MCS was employed to simulate the time-domain waveforms of these signals with different frequencies after propagating in the underwater environment; the results are shown in Figure 6. The focus was placed on calculating the time proportion within the 10–90% range of the rising edge to evaluate the relative stability of the signal rising edge. Typically, the 50% amplitude point of the time signal is defined as the reference trigger level; deformation of the signal’s rising edge can introduce timing error and jitter. The results are presented in Figure 7. It should be noted that in the actual implementation process, timestamp alignment needs to be additionally performed to ensure the accuracy of signal analysis.

Simulation results show that as frequency increases, the proportion of the 10–90% rising edge in total rising edge duration grows progressively. As seen in Figure 7a–d, as the frequency of the time signal increases, both jitter and edge diffusion become more significant. High-frequency time signals suffer more distortion because their smaller wavelength leads to larger phase changes from the same scattering-induced optical path disturbance. In contrast, low-frequency signals have stronger environmental robustness and better waveform stability. From another perspective, employing high-frequency timing signals allows for detecting and compensating for link noise interference with greater bandwidth.

**Figure 8:**
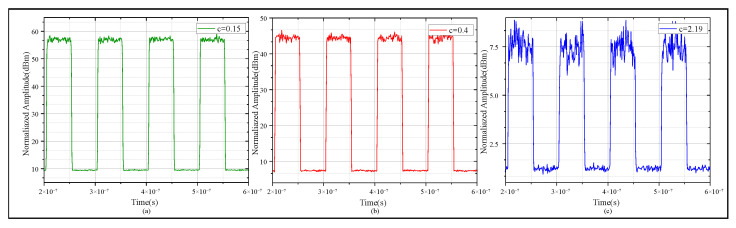
Performanceof the 10 MHz square wave signal transmitting under water conditions with different extinction coefficients. (**a**) Extinction coefficient is 0.15. (**b**) The extinction coefficient is 0.4. (**c**) The extinction coefficient is 2.19.

Furthermore, when simulating square wave propagation in water environments with different extinction coefficients, a typical time signal (10 MHz) was selected to analyze the influence of different attenuation and scattering characteristics on the signal’s time-domain properties; the results are shown in Figure 8. Figure 8a–c show the receiving 10 MHz square wave signals when the extinction coefficients are 0.15, 0.4, and 2.19, respectively.

**Figure 9:**
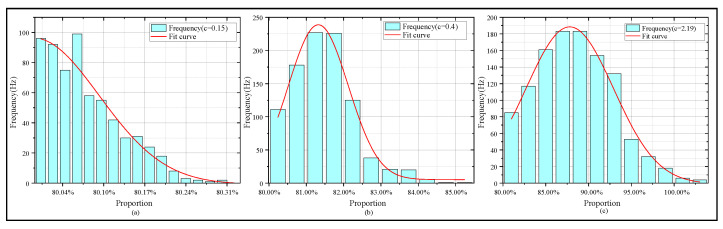
Histograms and fitting trends of 10 MHz simulated square wave signal’s 10–90% rising edge under water conditions with different extinction coefficients. (**a**) 10–90% rising edge statistics with extinction coefficient 0.15. (**b**) 10%–90% rising edge statistics with extinction coefficient 0.4. (**c**) 10–90% rising edge statistics with extinction coefficient 2.19.

We calculated the proportion of the 10–90% proportion in the total rising edge duration of the receiving square wave signal and plotted it in a histogram, as shown in Figure 9.

As shown in Figure 9a, with an extinction coefficient of 0.15, the fitting curve of the 10–90% rising edge time distribution is the most intact. As shown in Figure 9b, with an extinction coefficient of 0.4, edge widening happens, and the distribution concentrates mainly in the 81–82% range. As shown in Figure 9c, with an extinction coefficient of 2.19, the edge turns significantly blunt and signal jitter grows intense. This phenomenon hinders the accurate detection of clock edges and disrupts overall synchronization. We also built a test platform for UTFT experiment verification.

**Figure 10:**
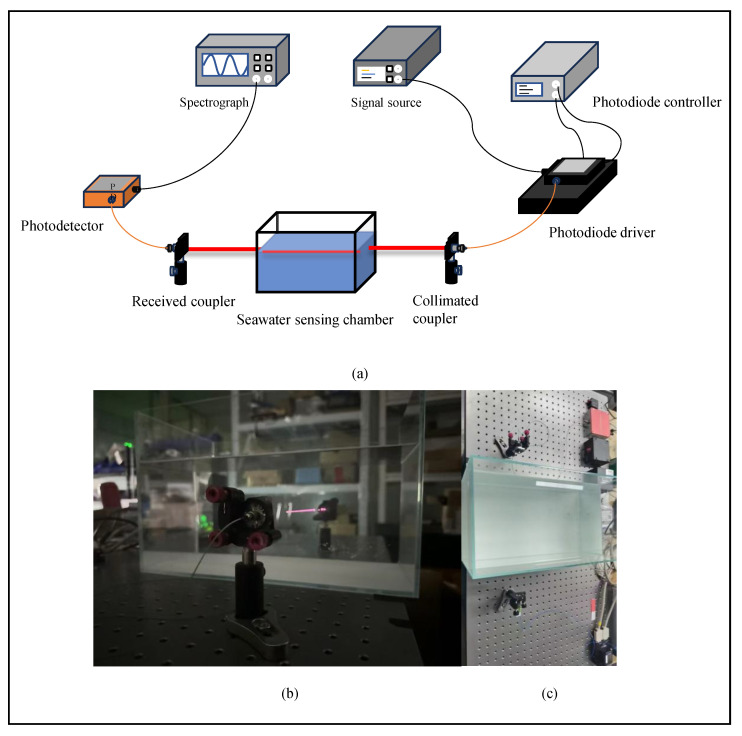
Experimental verification platform. (**a**) Experimental setup. (**b**) Optical sensing path configuration. (**c**) Panoramic environment.

The system construction of this experiment is shown in Figure 10. It is mainly used to verify the relationship between phase noise and turbidity under different turbidity derived from MCS. Four groups of testing signals with different frequencies, including 1 MHz, 10 MHz, 100 MHz, and 300 MHz, were set up in the experiment. These frequency signals were generated by a signal source with an adjustable frequency range. The signal was used to modulate a Laser Diode (LD). Direct modulation of the LD was achieved through a DC bias (Bias-T). An LD controller was used to provide current driving for the LD. Meanwhile, it performed high-precision temperature control of the LD (with a resolution of 0.001 °C) to ensure the LD output a stable optical signal. The modulated optical signal was incident into a seawater sensing chamber filled with kaolin suspending liquid via a fiber collimator. In the experiment, kaolin was used to prepare standard turbid liquid. The turbidity of the liquid was adjusted to achieve corresponding different extinction coefficients *c* m^−1^ (ranging from 0.04 to 0.40) [27]. At the receiving end, after passing through the seawater sensing chamber, the optical signal carrying scattering information about turbidity entered a photodetector through a receiving coupler. The photodetector was used to convert the received optical signal into an electrical signal. The converted electrical signal was collected by a spectrum analyzer, forming a complete sensing structure. Throughout the experiment, the output amplitude of the signal source, modulation depth, and incident optical power were kept constant. This ensures the comparability of experimental results under different turbidity conditions or different signal frequencies.

**Figure 11:**
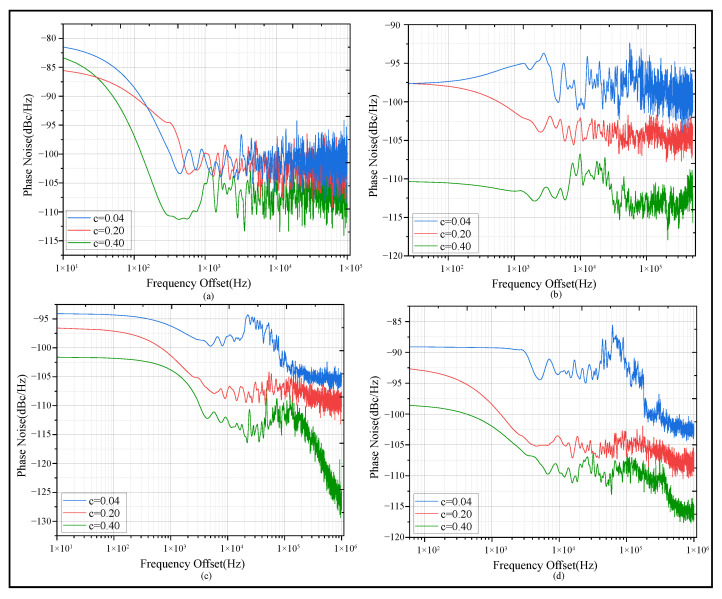
Phase noise versus increasing turbidity. (**a**) Phase noise corresponding to different extinction coefficients when the frequency is 1 MHz. (**b**) Phase noise corresponding to different extinction coefficients when the frequency is 10 MHz. (**c**) Phase noise corresponding to different extinction coefficients when the frequency is 100 MHz. (**d**) Phase noise corresponding to different extinction coefficients when the frequency is 300 MHz.

To verify the influence of the turbidity on signal stability and phase noise characteristics, this study conducted experimental measurements under different turbidity conditions. The spectral data of the signals at the receiving end were recorded under multiple frequencies (1 MHz, 10 MHz, 100 MHz, and 300 MHz), and phase noise was extracted from receiving signals. The empirical equation used was expressed as follows:

(5) $$\psi_{Noise}\left(f\right)=B\left(f\right)-A_{offset}-10*log_{10}\left(a*RBW\right)+b.$$

where, $\psi_{Niose}\left(f\right)$ represents the extracted phase noise, B(f) denotes the single-sideband spectrum data collected by the spectrograph, $A_{offset}$ stands for the peak power, RBW refers to the resolution bandwidth value, both *a* and *b* are constants determined by system parameters. The results are shown in Figure 11.

As Figure 11 shows, the blue phase noise curve corresponds to the condition of low turbidity (*c* = 0.04), where the received signal maintains high phase stability and the phase noise remains at a low level. The red phase noise curve represents the scenario where the turbidity is further increased (*c* = 0.20). The green phase noise curve denotes the results under the relatively high turbidity condition in the experiment (*c* = 0.40), with phase noise increasing significantly as turbidity rises. This observation can be explained by the fact that high-frequency signals, due to their higher temporal resolution, are more sensitive to minor perturbations such as scattering, making them prone to phase noise accumulation.

**Figure 12:**
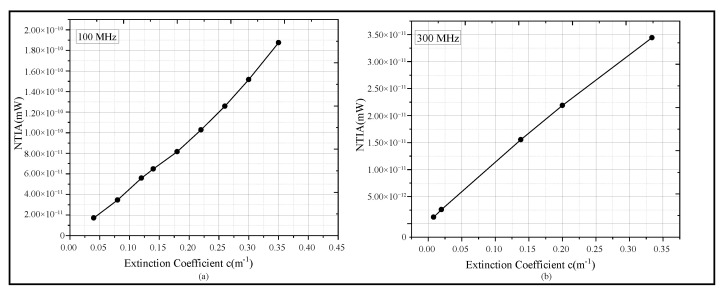
Fitting of NTIA results corresponding to different extinction coefficients in simulation and experiment. (**a**) The simulation results of the NTIA of phase noise when the extinction coefficient changes from 0.04 to 0.35. (**b**) The experimental results of the NTIA of phase noise when the extinction coefficient changes from 0.01 to 0.35.

Considering that it is relatively difficult to directly extract and accurately describe the relationship between phase noise and extinction coefficient, this study proposes and establishes a linear characterization model between the normalized trapezoidal integral area (NTIA) of phase noise in Figure 11 and turbidity. The comparison results between simulation and experiment are shown in Figure 12.

Figure 12a presents the fitting results of the simulation results at a frequency of 100 MHz, while Figure 12b shows the fitting results of the experiment results at a frequency of 300 MHz. A regression analysis was performed; the fitting equation derived from experimental data of Figure 12b is $y=1.0259110^{-11}\cdot x+7.779710^{-13}$. The results indicate that the Pearson correlation coefficient is 0.992, and the coefficient of regression $R^{2}=0.99839$.

Both the simulation and experimental results demonstrate that the NTIA of the phase noise characteristic exhibits a good linear relationship with the extinction coefficient *c*. This linear relationship holds true under the ideal conditions of numerical simulations and in actual experimental environments with kaolin added as a turbidity causing agent. This result verifies the rationality of the MCS model and provides a new quantitative index for predicting the performance of UTFT.

Based on the linear fitting relationship between NTIA and the extinction coefficient, feedforward prediction for UTFT systems can be realized. For instance, when planning a UTFT network, if the typical turbidity parameters of the target water area are known, the phase noise level and temporal stability of the system under different transfer distances can be estimated through the fitted extrapolation model. This enables pre-evaluation of the network’s feasibility and tolerance, while the NITA analysis method proposed in this study provides a convenient and reliable means for performance prediction of underwater optical TFS systems. Verified through mutual validation between simulations and experiments, this method exhibits strong applicability and robustness across different water environments. Such characteristics not only hold guiding significance for the design of underwater TFS links and provide a performance evaluation basis for high-speed optical communication systems such as underwater sensor networks, but also offer critical guidance for the construction and optimization of future UTFT networks. Specifically, it can help determine the optimal transmission distance range under specific water quality conditions, laying a theoretical foundation for the tolerance design, reliability improvement, and application promotion of UTFT systems.

## 4. Conclusions

Focusing on the UTFT issue, this study proposes and implements a simulation model based on the MCS, and conducts systematic verification through kaolin turbidity experiments. Firstly, the study analyzes the propagation characteristics of optical signals in different water types using the MCS model. Subsequently, it further investigates the influence of system parameters such as divergence angle, receiving aperture, and FOV on transfer performance, and finds that a small divergence angle and moderately restricted receiving parameters can effectively improve signal purity and frequency stability. In terms of frequency characteristics, both experiments and simulations show that high-frequency signals are more sensitive to turbidity and scattering, and more prone to cause phase noise accumulation. The analysis reveals the quantitative relationship between turbidity and the frequency transfer phase noise introduced thereby, and establishes a linear fitting model. This finding not only proves the accuracy of the MCS model but also provides a reliable tool for performance prediction.

In summary, the research findings of this paper first provide a theoretical basis and experimental support for the design and optimization of UTFT networks. Specifically, they enable the prediction of transmission performance under different water quality and distance conditions prior to network construction, which is of great significance for enhancing the robustness and tolerance of such networks. This study reveals quantitative effects of critical factors (water turbidity, system parameters, signal frequency) on UTFT performance, providing clear optimization directions. It provides theoretical and experimental support for practical UTFT applications and lays a foundation for scalable UTFT network design and tolerance optimization. Furthermore, these achievements also offer valuable reference for broader underwater optical communication applications, including marine exploration, thereby providing support for the advancement of related underwater communication systems. Future work will focus on verifying the model’s applicability in real marine environments, optimizing transmission and reception parameters to enhance long-distance UTFT stability, and exploring coordinated synchronization solutions for multi-node networks, aiming to promote the practical application of this technology in marine exploration.

## Figures and Tables

**Table 1:** Scattering, absorption, and extinction coefficients in different types of seawater.

Water Types	$\alpha /m^{-1}$	$\beta /m^{-1}$	$c/m^{-1}$
Clear water	0.114	0.037	0.151
Coastal water	0.179	0.219	0.398
Harbor water	0.366	1.824	2.190

## Data Availability

The data presented in this study are available on request from the corresponding author.

## Abbreviations

The following abbreviations are used in this manuscript:

AUV	Autonomous underwater vehicle
FOV	Field of view
H-G	Henyey–Greenstein
LBL	Long Baseline
LD	Laser diode
MCS	Monte Carlo simulation
NTIA	Normalized trapezoidal integral area
SNR	signal-to-noise-ratio
TDOA	Time difference of arrival
TFS	time–frequency synchronization
UTFT	Underwater time and frequency transfer
UWOC	Underwater wireless optical communication
